# A Microbial Piñata: Bacterial Endosymbionts of Trichomonas vaginalis Come in Different Flavors

**DOI:** 10.1128/mbio.01323-22

**Published:** 2022-08-15

**Authors:** Marina Ferrari de Aquino, Augusto Simoes-Barbosa

**Affiliations:** a School of Biological Sciences, University of Auckland, Auckland, New Zealand

**Keywords:** *Trichomonas vaginalis*, mycoplasma, symbiosis, endosymbiont, vaginal microbiome, sexually transmitted infections, trichomoniasis

## Abstract

The protozoan parasite Trichomonas vaginalis causes trichomoniasis, a prevalent human urogenital infection with significant morbidity that is commonly associated with vaginal dysbiosis. Exacerbation of T. vaginalis pathogenicity has been related to endosymbionts, including mycoplasma, and thought for a while to be solely attributable to Mycoplasma hominis. In a recent publication, Margarita and colleagues (https://journals.asm.org/doi/10.1128/mbio.00918-22) showed that endosymbiosis extends to a second species of mycoplasma known as “*Candidatus* Mycoplasma girerdii.” Those authors confirmed the strong association of T. vaginalis with both species of mycoplasma by reassessing clinical samples. Additionally, they showed that *in vitro* symbiosis of protozoa and bacteria resulted in the modulation of gene expression of T. vaginalis and enhancement of parasite cytoadhesion and hemolytic activity in culture assays. In this commentary, we portray T. vaginalis as a synergistically interacting multimicrobe organism—a “microbial piñata”—whose endosymbionts contribute significantly to the pathophysiology of this medically important protozoan parasite.

## COMMENTARY

The cervicovaginal microbiome (CVM) has an enormous impact on human sexual health and reproductive outcomes. However, compared to research on the gut microbiome, research on the CVM is highly underappreciated. With a dense microbial community of relatively low species complexity, with many of these microbes culturable in the laboratory, the CVM is an excellent tractable system for studying host-microbe interactions (1). Urogenital infection by the extracellular protozoan parasite Trichomonas vaginalis (i.e., trichomoniasis) and bacterial vaginosis (BV), a dysbiotic condition, are two leading examples of sexual diseases that are of concern due to their prevalence and reproductive-associated morbidities, to say the least (2, 3). Both infections are treated by metronidazole (MTZ), a prodrug that is selectively activated via intracellular reduction, electrons for which are provided through the activities of metabolic enzymes of these anaerobic microorganisms (2, 3).

**FIG 1:**
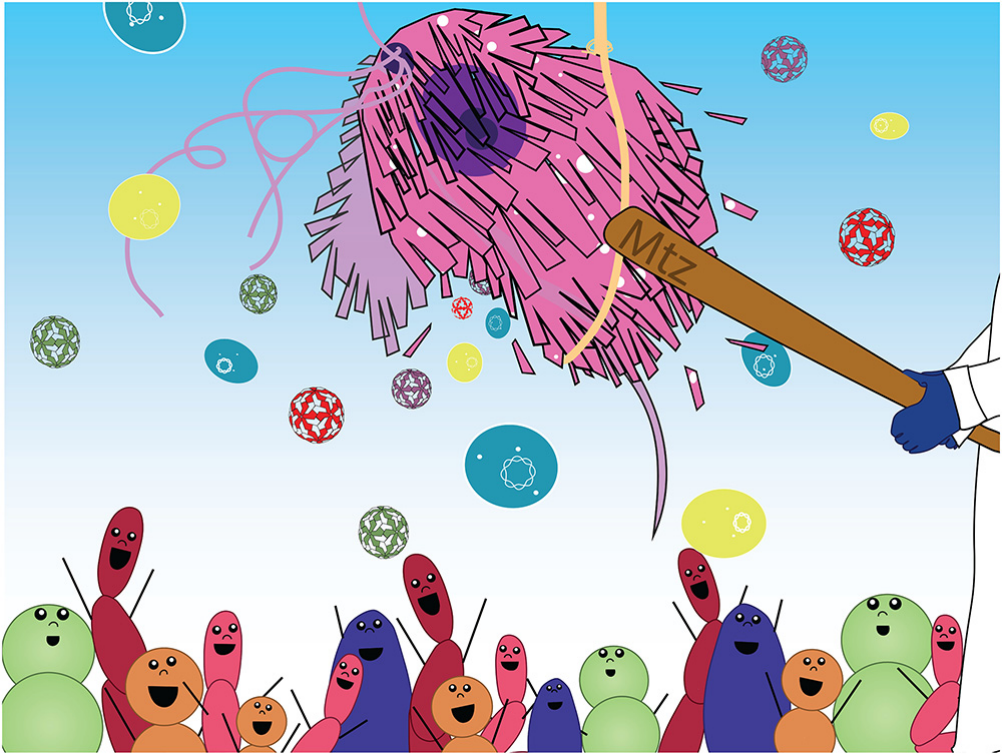
Trichomonas vaginalis portrayed as a piñata. The standard drug metronidazole, upon intracellular activation, binds to DNA and metabolic proteins, killing the parasite. The burst of protozoan cells releases variants of a specific family of RNA virus or Trichomonasvirus, represented by the geometric capsids in different colors, and two species of *Mycoplasma*, represented by the blue and yellow oval-shaped bacterial cells with circular DNA. The association of this infection with vaginal dysbiosis, a BV-like species-diversified cervicovaginal microbiome, is represented by bacterial cells of various colors at the bottom of the figure. T. vaginalis endosymbionts contribute to the pathobiology of trichomoniasis, as reviewed by Dessi et al. (7). This has now been expanded to a new species of mycoplasma endosymbiont by Margarita et al. (https://journals.asm.org/doi/10.1128/mbio.00918-22), the study that is the focus of this commentary.

Trichomoniasis is accompanied by a shift to vaginal dysbiosis in a clinical setting, and vaginal commensals have been shown to have significant effects on host and parasite responses in a laboratory setting (4). Furthermore, T. vaginalis is known for hosting two endosymbionts: a family of RNA viruses and *Mycoplasma* spp. (5). A recent article by Margarita et al. (https://journals.asm.org/doi/10.1128/mbio.00918-22), the focus of this commentary, depicts T. vaginalis as a carrier of two species of *Mycoplasma* that contribute to the pathophysiology of this protozoan, leading us to envisage T. vaginalis as a “microbial piñata” whereby MTZ kills the parasite while promoting mycoplasma dissemination on the urogenital mucosa (Fig. 1).

Mycoplasmas are tiny and ubiquitous parasitic bacteria of the class *Mollicutes* that lack cell walls and have minimal genomes, with many species known to parasitize various mucosal sites. Of interest to the CVM, Mycoplasma hominis is known to infect the urogenital tract and has been highly associated with trichomoniasis and BV (6). The group led by Pier L. Fiori has long been researching the relationship between T. vaginalis and mycoplasma and has revealed a very intricate three-way relationship (host-protozoa-endosymbiont) that implicates various aspects of the host response and parasite virulence (7).

Their latest research on this topic has culminated in their recent article by Margarita et al. A main driver of their study was the previously described strong association of trichomoniasis with the newly characterized species “*Candidatus* Mycoplasma girerdii.” This relationship was shown to be possibly even tighter than that to M. hominis, for which the study also confirmed the strong association of trichomoniasis with a BV-like CVM (8). Margarita et al. thought then to investigate the interactions of T. vaginalis with both species of *Mycoplasma* endosymbionts (M. hominis and “*Ca*. Mycoplasma girerdii”) in a laboratory setting for the first time. Before the experimentation, they searched for coinfections of T. vaginalis with mycoplasmas across clinical samples. When examining the 16S rRNA profile of vaginal swabs from patients that were positive for “*Ca*. Mycoplasma girerdii,” Maragarita and colleagues confirmed this tight association with T. vaginalis in 83% of the cases.

Furthermore, molecular typing revealed that more than half of the 75 clinical isolates of T. vaginalis were likely parasitized by both M. hominis and “*Ca*. Mycoplasma girerdii.” Considering the populational cohort of this particular study and others elsewhere, this observation suggested that T. vaginalis association with *M. hominis* and/or “*Ca*. Mycoplasma girerdii” is widespread and globally distributed and that the dual mycoplasma association may be more common than presumed. With that in mind, the authors measured the multiplicity of infection (MOI) in T. vaginalis isolates harboring either one mycoplasma species (T. vaginalis with *M. hominis* or T. vaginalis with “*Ca*. Mycoplasma girerdii”) or both (T. vaginalis with *M. hominis* and “*Ca*. Mycoplasma girerdii”), and their results brought up interesting surprises. “*Ca*. Mycoplasma girerdii” was much more abundant when it was the only mycoplasma species residing in T. vaginalis cells, i.e., T. vaginalis with “*Ca*. Mycoplasma girerdii” versus T. vaginalis with *M. hominis* and “*Ca*. Mycoplasma girerdii”. This was in sharp contrast to results with *M. hominis*, for which the MOI increased by ~10-fold above that for the inverse comparison (i.e., T. vaginalis with *M. hominis* and “*Ca*. Mycoplasma girerdii” versus T. vaginalis with *M. hominis*). Therefore, *M. hominis* prevails when T. vaginalis is naturally parasitized by both *Mycoplasma* species.

These findings led the authors to hypothesize that some type of intracellular competition may occur between the two *Mycoplasma* species when in symbiosis with T. vaginalis. To test this hypothesis, the authors established mycoplasma infections of T. vaginalis in laboratory cultures. They first removed the endosymbionts and reinfected the protozoan with either “*Ca*. Mycoplasma girerdii,” *M. hominis*, or both. The results of the *in vitro* symbiosis experiments agreed with the observations seen in the clinical samples, indicating that intracellular competition of the two *Mycoplasma* species within T. vaginalis leads to a decrease in the numbers of “*Ca*. Mycoplasma girerdii,” suggesting that *M. hominis* might exert strong pressure against “*Ca*. Mycoplasma girerdii” infection. Additionally, two alternative scenarios can be envisaged. First, T. vaginalis might be more susceptible to infection by *M. hominis* than that by “*Ca*. Mycoplasma girerdii,” at least *in vitro*. Second, this microbial association may possibly demand a different level of cooperation between the protozoan and its endosymbiont that is dependent on the species of *Mycoplasma*. In other words, is there a differential benefit to T. vaginalis when associated with one or another species of *Mycoplasma*?

Although Margarita et al. showed that T. vaginalis benefits from this association with a growth improvement, the *Mycoplasma* species present did not seem to make a difference. However, it is important to note that T. vaginalis grows in rich, complex media with 10% serum in the laboratory, a condition far from a natural infection. Thus, it is possible that a differential growth benefit may exist if under nutrient-restricted conditions. For example, Fiori's group has previously shown how the intertwined arginine dihydrolase pathways (ADH) of T. vaginalis and *M. hominis* are important for energy generation (7), genes for which “*Ca*. Mycoplasma girerdii” does not possess (9), and that the overconsumption of arginine could also benefit the parasite by diminishing nitric oxide production during an immune response (7). Therefore, their study paves the way for exploring the pathobiology of T. vaginalis in association with this newly characterized species of *Mycoplasma*, the comparative effects of single and dual mycoplasma associations, and how competition and cooperation might drive this endosymbiotic relationship.

A significant highlight of the work by Margarita et al. was undoubtedly the establishment of *in vitro* symbiosis from isogenic T. vaginalis strains. The authors could then make a sharp comparison of gene expression levels by RNA sequencing (RNA-seq). Here, they observed a significant overlap in the expression profiles of the mycoplasma-infected strains, biased to upregulation compared to the noninfected strain, making it hard to pinpoint the exact differences attributed to “*Ca*. Mycoplasma girerdii” or *M. hominis*. The authors mentioned the overall lack of modulation of the ADH genes but, as argued before, these expression profiles have come from T. vaginalis grown under optimal laboratory conditions. It is possible that differential gene expression will be more evident, and perhaps even more distinguishable, between T. vaginalis hosting either “*Ca*. Mycoplasma girerdii” or *M. hominis* once these strains are exposed to conditions closely resembling a natural infection. For instance, it was clear from their study that nutritional stress reduced the intracellular loads of mycoplasma, indicating that gene expression and cellular activity of T. vaginalis should be very adaptable to environmental conditions. Nevertheless, RNA-seq data revealed some critical information resulting from this symbiosis. There was clearly an upregulation of central metabolism with a shift from hydrogenosomal to cytosolic lactate and malate fermentation, along with an increase in amino acid catabolism, all of which help to explain the boost in the T. vaginalis growth rate when in symbiosis.

Another interesting observation was the upregulation of genes implicated in T. vaginalis pathogenicity, specifically, saposin-like protein (SAPLIP) and BspA. SAPLIPs are pore-forming proteins potentially involved in hemolysis by T. vaginalis (10). The leucine-rich repeat-containing BspA proteins of T. vaginalis (TvBspA) are surface proteins that have been implicated in the physical interaction of T. vaginalis with other cells (10). TvBspA expression was found to respond to mycoplasma infection, with some level of specificity to the mycoplasma species. It is a difficult task to draw a conclusion from such a massively expanded gene family (~900 members). However, 8 of the 11 TvBspA proteins previously found to be more abundant in highly adherent versus lowly adherent T. vaginalis strains (10) were also upregulated in response to mycoplasma. Knowing that cytoadhesion and cytolysis are central to the pathogenicity of T. vaginalis and are strongly co-related (11), Margarita and colleagues sought to evaluate whether the protozoa-bacteria endosymbiosis could modulate T. vaginalis pathogenicity. Although a cause-effect relationship between the expression of these virulence factors (i.e., SAPLIP and/or TvBspA) and pathogenicity was not established in this study, the authors demonstrated that this symbiotic association results in a phenomenal enhancement of hemolysis and cytoadhesion by T. vaginalis.

This is one of a few studies to question the reductive “pathogen-in-isolation” approach traditionally applied to understand the pathobiology of infectious diseases. Complex relationships among the host, pathogen, and microbiota are very influential to the disease outcomes. These multimicrobe associations within the host are implicated in comorbidities and might even drive microbiome disturbances in the long term. Thus, microorganisms that naturally coexist with a pathogen, i.e., commensals and endosymbionts, can no longer be considered simple spectators. The CVM and trichomoniasis make an excellent study case for such interactions (Fig. 1). Further understanding of the biology of the T. vaginalis-*Mycoplasma* relationship will be accelerated by the achievements of this study, particularly after the establishment of *in vitro* symbiosis with isogenic T. vaginalis strains.
